# Highly Sensitive Electrochemical Aptasensor for Detecting the VEGF_165_ Tumor Marker with PANI/CNT Nanocomposites

**DOI:** 10.3390/bios11040114

**Published:** 2021-04-09

**Authors:** Yunjeong Park, Min-Sung Hong, Woo-Hyuk Lee, Jung-Gu Kim, Kyunghoon Kim

**Affiliations:** 1 School of Mechanical Engineering, Sungkyunkwan University (SKKU), Suwon 16419, Korea; djy828@skku.edu; 2 School of Advanced Materials Science and Engineering, Sungkyunkwan University (SKKU), Suwon 16419, Korea; smith803@skku.edu (M.-S.H.); picohiyo@skku.edu (W.-H.L.)

**Keywords:** aptasensor, aptamer, polyaniline (PANI), carbon nanotube (CNT), nanocomposite, electrochemical sensor, vascular endothelial growth factor (VEGF_165_)

## Abstract

Sensing targeted tumor markers with high sensitivity provides vital information for the fast diagnosis and treatment of cancer patients. A vascular endothelial growth factor (VEGF_165_) have recently emerged as a promising biomarker of tumor cells. The electrochemical aptasensor is a promising tool for detecting VEGF_165_ because of its advantages such as a low cost and quantitative analysis. To produce a sensitive and stable sensor electrode, nanocomposites based on polyaniline (PANI) and carbon nanotube (CNT) have potential, as they provide for easy fabrication, simple synthesis, have a large surface area, and are suitable in biological environments. Here, a label-free electrochemical aptasensor based on nanocomposites of CNT and PANI was prepared for detecting VEGF_165_ as a tumor marker. The nanocomposite was assembled with immobilized VEGF_165_ aptamer as a highly sensitive VEGF_165_ sensor. It exhibited stable and wide linear detection ranges from 0.5 pg/mL to 1 μg/mL, with a limit of detection of 0.4 pg/mL because of the complementary effect of PANI/CNT. The fabricated aptasensor also exhibited good stability in biological conditions, selectivity, and reproducibility after several measurement times after the dissociation process. Thus, it could be applied for the non-invasive determination of VEGF, in biological fluid diagnosis kits, or in an aptamer-based biosensor platform in the near future.

## 1. Introduction

Vascular endothelial growth factor (VEGF_165_), a glycolated protein belonging to the platelet-derived growth factor family of the cystine-knot growth factors, is essential for vascular development [1]. It is also active in angiogenesis, vasculogenesis, and endothelial cell growth, causing endothelial cell proliferation, promoting cell migration, inhibiting apoptosis, and inducing blood vessel permeabilization [2,3,4]. However, when tumor lymphatic vessels and metastasis of cancer are induced, abnormally fast growth and division of tumors can promote overexpression of VEGF_165_ [5]. A high level of VEGF_165_ can be regarded as a predictor of different cancers, such as brain, lung, breast, lymphoma, and gastrointestinal tumors [6,7,8]. Therefore, VEGF_165_ has been recently suggested as a promising biomarker for diagnosing cancers [9,10,11]. In addition, VEGF-mediated eye disease is related to VEGF_165_, and such patients have a VEGF concentration ranging from 74.5 to 521.6 pg/mL [12]. For the patient of VEGF drug treatment, there are a few significant studies on the detection of VEGF_165_ levels in bio samples [13,14,15,16]; however, more accurate, reliable, and stable detection methods with higher sensitivity are still required.

Lately, aptamers have received attention in the field of biosensor due to their remarkable binding affinities and stability [17,18]. Aptamers contain an artificial oligo-nucleic acid or peptide molecule, which is a single-stranded DNA (ssDNA) or RNA [19]. Aptamers are also called “chemical antibodies” due to their artificial process in vitro based on the systematic evolution of ligands by the exponential enrichment (SELEX) technique. Unlike the production of commercial antibodies, which depends on the derivation of an animal immune system, the SELEX process can synthesize aptamers for non-immunogenic and toxic targets [20]. For this reason, they can replace antibody bioassays because aptamers are easy to synthesize, highly resistant to denaturation, and low cost [21]. Due to their high specificity and affinity, which came from their ability of folding upon binding with their target molecule, aptamers can be selected in vitro for any given target, ranging from small molecules to large proteins and even cells, which can make various aptamer-based biosensors [22,23]. Biosensors made with aptamers based on various nanomaterials have been designed to detect very low concentrations of their targets [24,25,26]. However, it is still challenging to optimize the anchor of an aptamer on nanomaterials for aptamer-based biosensors (aptasensors).

Carbon nanotubes (CNTs) are among the most widely researched one-dimensional materials due to their excellent mechanical, heating, electrical, and optical properties [27]. Especially, CNTs are suitable for making electrode materials: because of their high electrical conductivities and diverse surface modification, CNTs provide an enhanced electrocatalytic reaction and good stability [28,29]. Because CNTs facilitate electron transfer between electroactive species and the electrode, they offer great promise for fabricating chemical sensors or biosensors [27,30]. Therefore, many studies have achieved high stability and electrical properties of CNTs by combining them with various effective materials. Not only that, but significant progress also has been made on nanocomposites with the synthesis of conductive polymer and CNTs, which show excellent potential to apply in various fields because of their outstanding physical and chemical characteristics originating from their small dimensions and high surface areas [31,32]. Among the conducting polymers, polyaniline (PANI) is a unique conjugated polymer that can be used for specific applications when it is combined with nanomaterials [33,34]. The incorporation of CNT with PANI acts as a network film that leads to a high carrier mobility and high surface-to-volume ratio; it also minimizes the loss of signal intensity because of the direct path for charge/ion transportation while offering improved sensitivity [35,36].

The objective of this study was to introduce a simple and highly sensitive electrochemical biosensor to detect VEGF_165_ by incorporating PANI and CNT with an anti-VEGF_165_ RNA aptamer on a screen-printed carbon electrode (SPCE). Such a biosensor can achieve a simple and accurate VEGF_165_ detection as a powerful platform for bioanalysis and clinic diagnostic application. It can be used in various flexible devices due to the polymeric property of the PANI/CNT nanocomposite.

## 2. Materials and Methods

### 2.1. Materials

CNTs (purity > 95%, diameter ~1.5 nm, length = 1–5 μm) were commercially purchased from NanoLab (Waltham, MA, USA). H_2_SO_4_, HNO_3_, ammonium persulfate (APS), aniline, and vascular endothelial growth factor (VEGF_165_) were purchased from Sigma-Aldrich (St. Louis, MO, USA). Deionized (DI) water was used for PANI/CNT synthesis and washing purposes. SPCE was obtained from EG Tech (Bucheon, Korea). Anti-VEGF_165_ RNA aptamer was obtained from Bioneer Co. (Daejeon, Korea). The aptamer sequence was as follows: 5′-NH_2_-AUG CAG UUU GAG AAG UCG CGC AU-3′.

### 2.2. Fabrication of PANI/CNT Nanocomposite on SPCE

Carboxylic acid functionalized CNTs were prepared according to the conventional oxidation method [37]. Pristine CNTs (100 mg) were suspended in 24 mL mixture of concentrated H_2_SO_4_/HNO_3_ (3:1, *v*/*v*). They were then sonicated in a water bath at 35–45 °C for 24 h. After cooling to room temperature, the suspension was then filtered with 200 nm porous filter membrane (PTFE; Millipore). Filtrated materials were washed several times with DI water until a neutral pH was obtained. Collected materials were then dried in a vacuum oven at 50 °C for 12 h.

Functionalized CNTs were suspended in DI water and sonicated in a water bath at 25 °C for 30 min to prepare a 0.5 mg/mL CNT solution. A suspension (45 μL) of functionalized CNTs was added to 1 M HCl (6 mL) and sonicated in a water bath at 25 °C for 15 min. After adding 9 mg of APS, the mixture was sonicated for 15 min. The mixture was then placed in a refrigerator at 5 °C for 30 min. After 6.5 μL of aniline was dropped into the above mixture, the mixture was then dropped on the SPCE surface and dried overnight at 40 °C for further characterization. PANI-only nanocomposites were also prepared with the above method without adding CNTs.

### 2.3. Immobilization of Aptamer on PANI/CNT Nanocomposite

Aptamer stock solution was diluted with diethylpyrocarbonate (DEPC)-treated water (0.1 wt%) and stored at −20 °C in a freezer before use. To attach the anti-VEGF_165_ RNA aptamer onto the surface of the PANI/CNT nanocomposite, a mixture of anti-VEGF_165_ RNA aptamer (10 nM) and 4-(4,6-Dimethoxy-1,3,5-triazin-2-yl)-4-methyl-morpholinium chloride (DMT-MM) solution (1 wt%; 40 μL) was incubated with the SPCE surface for 12 h. DMT-MM is an efficient condensing agent. It was prepared according to the published description [38]. The condensing process on the surface can be expressed as follows [32]:

PANI/CNT nanocomposite − COOH + H_2_N − Anti-VEGF_165_ RNA aptamer ↓ PANI/CNT nanocomposite − CONH − Anti-VEGF_165_ RNA aptamer

**Figure 1:**
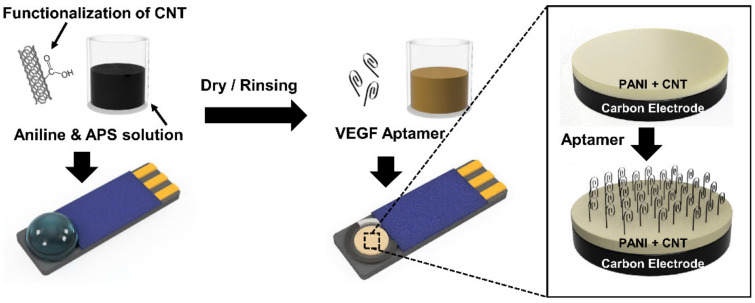
Schematic illustration of the preparation of the polyaniline/carbon nanotube (PANI/CNT) nanocomposite and the assembled VEGF_165_ aptamer on the sensor surfaces for the VEGF sensors.

Afterwards, the SPCE was rinsed with DI water and dried with a stream of nitrogen gas (Figure 1).

### 2.4. Characterization of the Biosensor

Surface morphology was observed using a scanning electron microscope (SEM; JSM-7600F, Jeol Ltd., Tokyo, Japan). For the surface elements analysis, Fourier-transform infrared (FT-IR) spectra of the PANI/CNT were obtained using an IFS-66/S FT-IR spectrometer (Bruker, Ettlingen, Germany) and an X-ray photoelectron spectroscope (XPS) recorded with ESCALAB250 equipment (Thermo Electron, Waltham, MA, USA) and an Al Kα monochromatic X-ray source (hv = 1486.6 eV).

### 2.5. Electrochemical Analysis to Evaluate the Sensitivity of the Biosensor

For a three-electrode system of the SPCE, a working electrode (WE) and a counter electrode (CE) were printed with carbon ink. Silver (Ag) ink was used as the reference electrode (RE). The three-electrode system of SPCE was connected to an electrochemical apparatus (VSP 300, Bio-Logic SAS, Seyssinet-Pariset, France). All electrochemical tests were performed in 10 mM (Fe(CN)_6_)^3−/4−^ as the redox agent in a PBS buffer solution, resulting in a charge transfer resistance. Cyclic voltammetry (CV) tests were conducted at the voltage range of −0.2 V to 0.7 V with a scan rate of 50 mV/sec. Electrochemical impedance spectroscopy (EIS) was carried out with an amplitude of 20 mV in a frequency range of 100 kHz to 10 MHz. The potential was set to be 0.2 V (vs. Ag/AgCl) for all EIS measurements. Impedance plots were interpreted on the basis of equivalent circuits using a suitable fitting procedure with ZsimpWin software (ZsimpWin 3.20, EChem Software, Ann Arbor, MI, USA). Differential pulsed voltammetry (DPV) tests were performed at the voltage ranging from −0.2 V to 0.4 V with A pulse height of 50 mV and pulse width of 50 ms. Step height and step time of the DPV tests were set at 5 mV and 500 ms, respectively. The selectivity of the modified biosensor was compared using other bio-proteins, such as human serum albumin (HSA), human immunoglobulin (HIgG), lipase (Lip), and lysine (Lys), which were used as interference molecules.

## 3. Results and Discussion

### 3.1. Characterization of the PANI/CNT Nanocomposite

**Figure 2:**
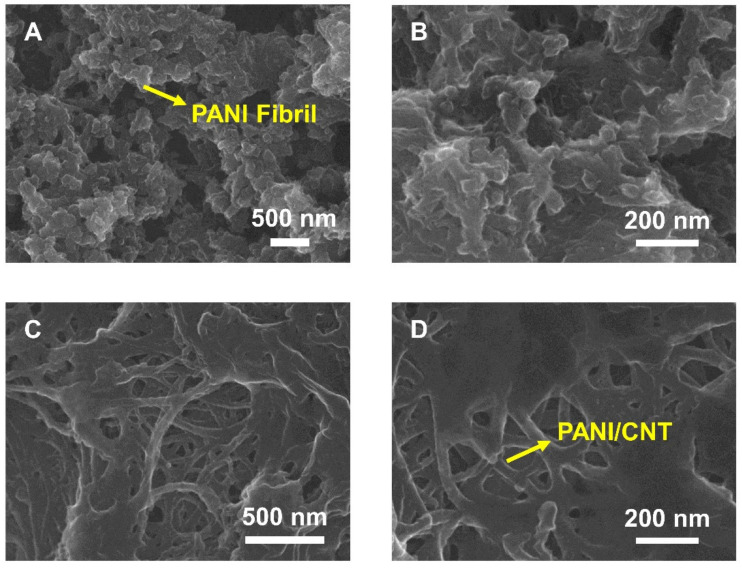
Scanning electron microscope (SEM) images of (**A**) PANI and (**B**) an enlarged image of PANI. (**C**) The PANI/CNT nanocomposite and (**D**) an enlarged image of the PANI/CNT nanocomposite.

Acid-treated CNTs have high dispersity in an aqueous environment due to various functional groups such as hydroxyl and carboxyl groups. These functional groups can act as active sites that are capable of attaching some anilinium ions by electrostatic attraction. Thus, polymerization of aniline on the active sites of CNTs make the PANI/CNT nanocomposites. For comparison, SEM images of PANI (Figure 2A,B) and PANI/CNT (Figure 2C,D) were obtained to characterize their morphologies. The PANI-modified electrode showed porous nano-fibril structures [39,40]. On the other hand, the PANI/CNT-modified electrode showed individual fibers, indicating that the PANI covered the surface of the CNTs because the active sites of the CNTs could act as a backbone for PANI [36,39,40].

**Figure 3:**
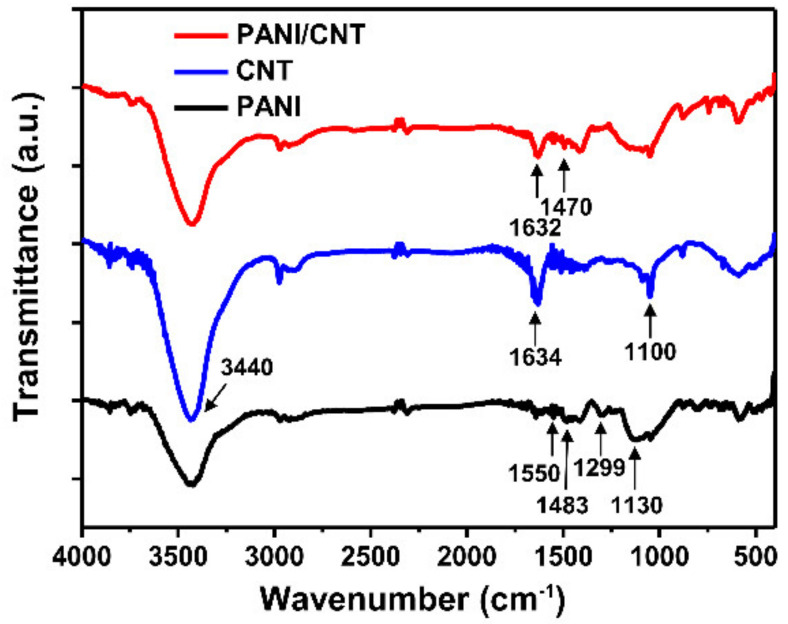
Fourier-transform infrared spectroscopy (FT-IR) spectra of the PANI, CNT, and PANI/CNT nanocomposites.

FT-IR spectra were obtained to determine the chemical constitutions of the PANI/CNT nanocomposite. As shown in Figure 3, PANI had distinct peaks at 1550cm^−1^, 1483 cm^−1^, and 1299 cm^−1^, representing the quinoid ring, benzenoid ring, and C–N stretching mode of the second arylamine, respectively, indicating an oxidation state of emeraldine salt (ES) of PANI. In addition, a strong peak at around 1130 cm^−1^, representing an electron-like band, was found for PANI [36]. CNT had large peaks at 3440 cm^−1^, 1634 cm^−1^, and 1100 cm^−1^, representing O–H stretching from the carboxyl group (O=C–OH and C–OH), the stretching of C=C in the quinoid ring, and C–O stretching vibrations, respectively. The PANI/CNT nanocomposite showed obvious peaks at 1632 cm^−1^ and 1470 cm^−1^, representing the quinoid ring of the C=C structure of the CNT and benzenoid ring of the ES state of the PANI chains, similar to the FR-IR spectra of the PANI/CNT. These results indicate that the surface of the CNTs was coated with PANI to successfully form the PANI/CNT nanocomposite.

### 3.2. Characterization of Biosensor Based on SPCE

**Figure 4:**
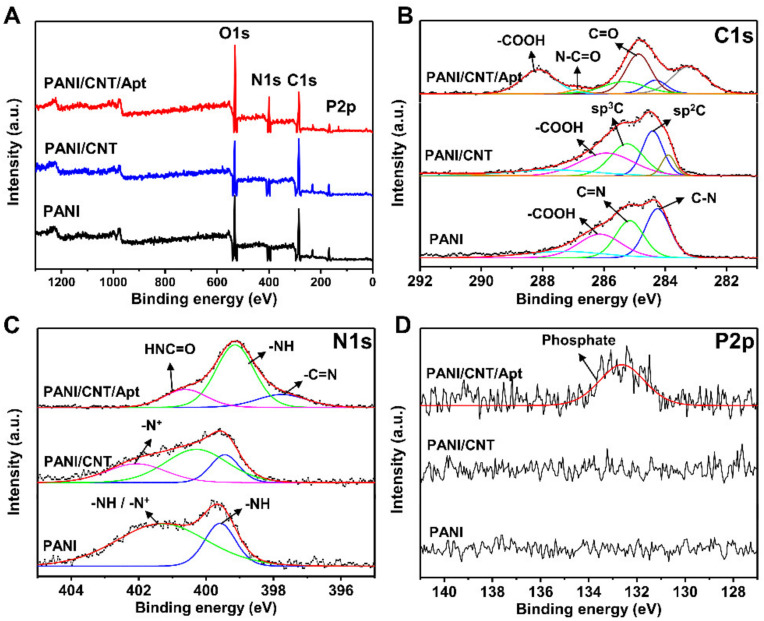
X-ray photoelectron spectroscopy (XPS) spectra of the modified electrode surfaces with PANI, PANI/CNT and PANI/CNT/Apt. (**A**) XPS wide spectra of the modified surfaces. (**B**) High-resolution XPS spectra of C1s, (**C**) N1s, and (**D**) P2p.

To characterize the modification of the SPCE electrode with the PANI/CNT and aptamer, deconvoluted XPS spectra of wide scan, C1s, N1s, and P2p were obtained, as shown in Figure 4. In the wide scan (Figure 4A), the N and O peaks were increased while the peaks of C decreased after aptamer immobilization due to the configuration of the aptamer with higher compositions of N and O but a lower composition of C. In addition, the P2p peak at 133 eV was found as a strong proof of successful immobilization of aptamers [41]. As shown in Figure 4B, the C1s spectrum had peaks similar to the PANI layer and PANI/CNT layer that appeared at 286.13 eV (–COOH group), 285.28 eV (sp^3^C and C=N bond), and 284.48 eV (sp^2^C and C–N bond). After the electrodes were modified with aptamers (PANI/CNT/Apt), the N–C=O peak corresponding to covalent bonding between the PANI/CNT and aptamer and C=O peak of the aptamer component newly appeared at 286.88 eV and 285.33 eV, respectively. In addition, the –COOH group peak shifted slightly to a higher energy at 288.15 eV. As shown in Figure 4C, N1s spectrum of PANI also had two peaks at 399.48 eV (–NH) and 401.38 eV (–NH and –N^+^). When PANI was combined with CNT, it showed a peak at 402.88 eV (–N^+^), indicating a higher binding energy. The PANI/CNT/Apt layer exhibited three new peaks at 400.68 eV (HNC=O), 399.18 eV (–NH), and 397.958 eV (C=N), indicating that a covalent bond was formed between the COOH group of the PANI/CNT and the NH_2_ of aptamer [41]. Moreover, the P2p spectrum of the PANI/CNT showed no peak, while the PANI/CNT/Apt layer showed a phosphate peak at 132.73 eV due to the aptamer molecules (Figure 4D) [41]. These results demonstrated that the PANI/CNT/Apt nanocomposite was successfully synthesized.

### 3.3. Electrochemical Property of the Biosensor Based on an Aptamer with a PANI/CNT Nanocomposite

**Figure 5:**
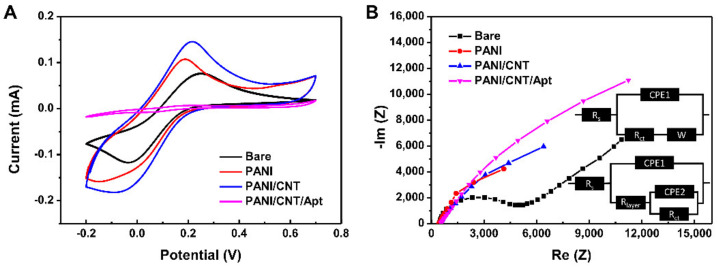
(**A**) Cyclic voltammetry (CV) at scan rate of 50 mV/sec and (**B**) the electrochemical impedance spectroscopy (EIS) at 0.2 V (vs. Ag/AgCl) in the frequency range of 100 kHz to 10 MHz spectra in 10 mM (Fe(CN)_6_)^3−/4−^ as the redox PBS buffer solution measured on PANI, PANI/CNT, and PANI/CNT/Apt-modified electrodes and unmodified SPCE. The inset is the electric circuit compatible with the Nyquist diagrams for the modified electrodes. R_s_: Electrolyte resistance; R_ct_: Charge transfer resistance; CPE1: Constant phase element; and W: Warburg diffusion impedance.

To elucidate the effect of the PANI/CNT film, the electrochemical properties of the composite film were evaluated with CV and EIS measurements in a test solution. According to the voltammogram results, which is shown in Figure 5A and Figure S2, the PANI, PANI/CNT, and PANI/CNT/Apt nanocomposite clearly had different electrochemical behaviors. After modification of the bare electrode with the PANI or PANI/CNT nanocomposite, the oxidation peak potential of the ferricyanide was increased due to its electrical conductivity. However, the PANI/CNT electrode had a higher conductivity than the PANI electrode because of the excellent electronic properties of the CNTs. The PANI/CNT nanocomposite clearly demonstrated stronger peaks, indicating that the nanocomposite had more electroactive sites than the PANI electrode. However, after the aptamer immobilization reaction, the redox peak of the PANI/CNT/Apt was dramatically decreased compared to that of PANI or PANI/CNT. This result indicates that the charge transfer resistance was increased because immobilization of the aptamers on electrode surface blocked the path of the redox reaction substance.

Differences in the modified electrodes were also determined by EIS test. The results are shown in Figure 5B. In the EIS plots, the bare electrode shows the property of a diffusion-limited process. On the other hand, the modified electrodes revealed semicircle curves, indicating that the electron transfer between (Fe(CN)_6_)^3−/4−^ and the electrode was blocked by the PANI, PANI/CNT, and PANI/CNT/Apt layers on the electrode [16]. The circuits are described as two modes: electrolyte resistance (R_s_), charge transfer resistance (R_ct_), layer (coating) resistance (R_layer_); CPE1 is the dielectric strength of the layer and solution absorbed by the layer and electric double layer at the electrolyte/substrate interface (CPE2) used for interface capacitance and Warburg diffusion impedance (W) at a low frequency [42,43]. In the EIS test results, the modified electrodes show incomplete semi-circle features. There are two reason for the incomplete semi-circle: the frequency range of the EIS tests and their surface characteristics. The EIS results of the modified electrodes need a few more points at the low frequency region for a complete semi-circle. However, it takes much more time to obtain the points in the low frequency region; therefore, generally, the frequency range is limited from 100 kHz to 10 MHz in the EIS test. For this reason, the EIS tests are used a simulation programs to obtain the calculated values from the tests. As shown in Figure S1, comparison of the R_ct_ values could indicate resistance of the specimen’s surface. The R_ct_ of the PANI-coated electrode of SPCE was recorded to be 14,240 Ω. The addition of CNT to PANI resulted in a reduction of R_ct_ to be 9851 Ω, which is due to the high electrical conductivity of the CNT. After immobilization of aptamers, the R_ct_ was dramatically increased to 46,170 Ω because the aptamer blocks the active sites on the nanocomposite surface for charge transfer. In addition, electrostatic repulsion between the negatively charged aptamer and solution-phase (Fe(CN)_6_)^3−/4−^ redox agent leads to suppressing the chemical reaction on the electrode surface [15]. As a result, in CV and EIS characterization, the Faraday currents and charge transfer resistance changes were consistent.

### 3.4. VEGF_165_ Detection and Monitoring

**Figure 6:**
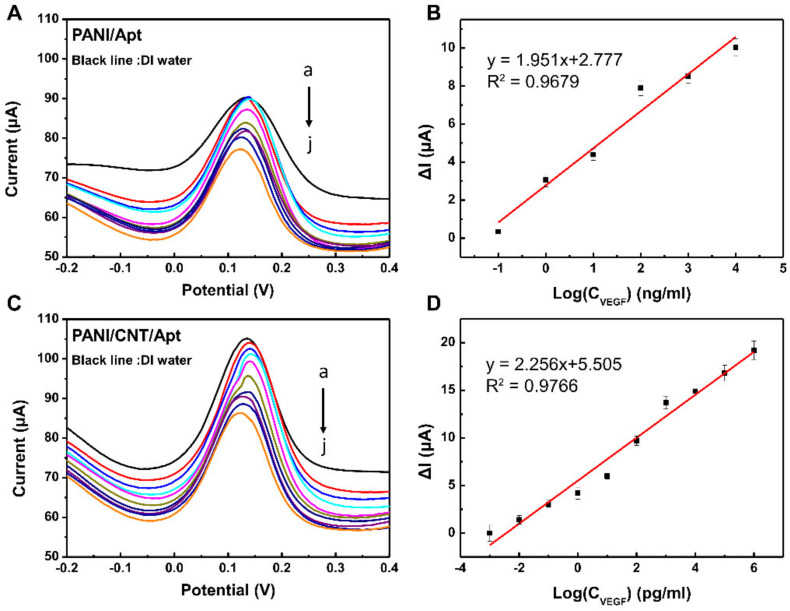
(**A**,**C**) Differential pulse voltammetry (DPV) plot, at range from −0.2 V to 0.4 V with a 5 mV height and 50 ms pulse width (step height: 5 mV and step time: 500 ms), of VEGF_165_ at different concentrations (from a to j): (reference black line: DI water) 10 fg/mL, 100 fg/mL, 1 pg/mL, 10 pg/mL, 100 pg/mL, 1 ng/mL, 10 ng/mL, 100 ng/mL, 1 μg/mL, and 10 μg/mL under the optimized conditions on the PANI/Apt- and PANI/CNT/Apt-modified electrodes. (**B**,**D**) Peak currents vs. log VEGF concentrations from the DPV plots.

Under an optimized experimental condition (described in the experimental section), analytical calibration was performed using the DPV test. Results are presented in Figure 6. As shown in Figure 6A, after incubating the PANI aptasensor, an enhanced peak current (I_peak_) was observed with decreasing concentration of VEGF_165_ due to the mass transfer limiting of (Fe(CN)_6_)^3−/4−^ to the electrode surface by binding with VEGF_165_ molecules. There was a linear relationship between I_peak_ and the logarithm of the VEGF_165_ concentration (C_VEGF_) in the range from 10 fg/mL to 1 μg/mL (I_peak_ = 2.776 + 1.951logC_VEGF_ (R^2^ = 0.968), R = correlation coefficient), with a limit of detection (LOD) of 0.7 ng/mL (3σ/S).

For comparison, the sensing performance of the modified electrode with PANI/CNT is shown in Figure 6B. The PANI/CNT aptasensor showed significantly decreased response currents, with a linear regression equation of I_peak_ = 5.505 + 2.25logC_VEGF_ (R^2^ = 0.977). It showed an excellent sensitivity with an LOD of 0.4 pg/mL (3σ/S) because the electrode modified with PANI/CNT had a large specific area and electron transfer rate favorable for signal transmission. In addition, the peak current of the bare PANI/CNT electrode is higher than that of the PANI electrode due to the same reasons. These results confirmed that the PANI/CNT-modified electrode could enhance the electrochemical signal and contribute to the high performance in VEGF_165_ detection. Besides, the PANI/CNT aptasensor showed comparable or better performance than other reported VEGF_165_ biosensors (Table 1). Considering the design of a diverse aptamer for a biosensor and the simple preparation process of the PANI/CNT-modified electrode, the high-performance of the PANI/CNT aptasensor fabricated in this study would have wide bio-applications.

### 3.5. Selectivity

**Figure 7:**
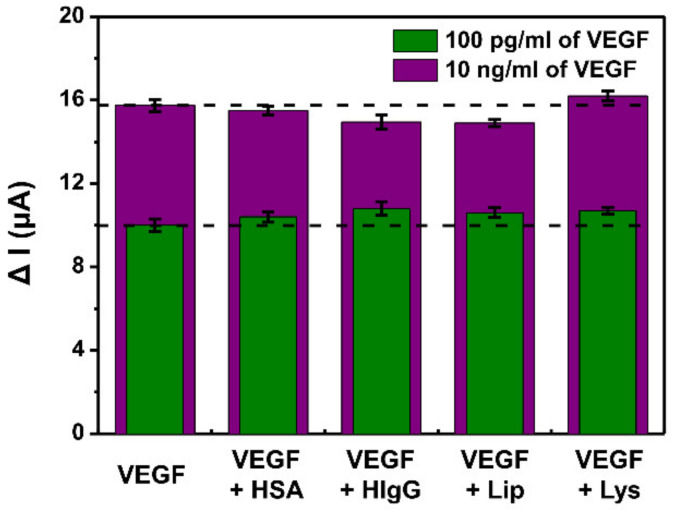
Selectivity of the PANI/CNT/Apt-modified electrodes shown in ΔI scales for VEGF_165_ in the presence of 100 times of HSA, HIgG, Lip, and Lys as the interfering species. The concentrations of VEGF_165_ are 100 pg/mL and 10 ng/mL and the interfering species are 10 ng/mL and 1 μg/mL.

Selectivity results of the PANI/CNT/Apt nanocomposite in sensing response to VEGF_165_ compared to other bio-proteins at the certain concentrations are shown in Figure 7. To evaluate the selectivity of the proposed aptasensor, some biomolecules, such as HSA, HIgG, Lip, and Lys, were used as interference molecules, which is the major nonspecific binding constituents of human serum blood. This process was performed to evaluate the selectivity to VEGF_165_ of the PANI/CNT/Apt aptasensor within the interfering agents. The base concentration of the VEGF_165_ was selected as 100 pg/mL and 10 ng/mL, and the concentration of the interference proteins was applied to 100 times higher than the base VEGF_165_ concentrations. As clearly shown in Figure 7, the results showed a negligible effect on the main signal. For example, HIgG resulted in only about a 0.5% error in the detection of VEGF_165_. This was because the physical adsorption of HIgG onto the surface-modified electrode can be easily washed away by the PBS rinsing. As expected, the proposed aptasensor possessed a high sensitivity for VEGF_165_ due to a high affinity and specificity of the aptasensor toward the presence of specific nucleic acid sequences in their structures that could bind a specific target.

Intra- and inter-reproducibility of the proposed aptasensor were also considered. The same as with the selectivity tests, the electrodes were rinsed in a PBS solution. The intra reproducibility was estimated by measuring 100 pg/mL of VEGF_165_ with the same aptasensor. The relative standard deviation (RSD) for a series of five experiments was found to be 5.6%. The inter reproducibility was evaluated with five difference aptasensors for determining 100 pg/mL of VEGF_165_. The RSD was calculated to be 6.8%. These results showed that the proposed aptasensor exhibited high analytical performance in terms of sensitivity, selectivity, reproducibility, stability, and low detection limit.

## 4. Conclusions

In conclusion, we successfully fabricated a highly sensitive VEGF_165_ detection sensor for cancer diagnosis. The sensor was assembled from a PANI/CNT nanocomposite by polymerization of APS and aniline at the CNT edge site with simultaneous film deposition. It exhibited high sensitivity for VEGF_165_ sensing with an excellent detection limit of 0.4 pg/mL (3σ/S) compared to other reported biosensors. It also showed highly selectivity in the presence of other proteins. Such an excellent sensing performance was ascribed to the complementary effects of the PANI/CNT surface area, effective network channels of the PANI/CNT, and the highly selective reaction of the aptamer. The film has the advantages of a simple preparation, high sensitivity, small size, and the possibility of modification by changing the aptamer for sensing other bio-molecules. Thus, it has potential for use in highly effective and sensitive bio-sensor devices in the future.

## Figures and Tables

**Table 1:** Comparison of the VEGF sensor based on the nanomaterial-modified electrodes.

Modified Electrode	Detection Method	Linear Range (pg/mL)	LOD (pg/mL)	Reference
GE/GO/ssDNA/PLLA NP	DPV	50.0–1.0 × 10^5^	50	Pan et al. 2017 [11]
PPy-NDFLG-FETs/Apt	FET	4.5–4.5 × 10^5^	4.5	Kwon et al. 2012 [13]
GE/Apt/MB-Abs	nFIS	5.0–1.0 × 10^3^	401.0	Qureshi et al. 2015 [14]
SPE/OMC-Au_nano_/Apt	EIS	10.0–300.0	1.0	Tabrizi et al. 2015 [15]
GE/Thiolated Apt	SWV	150.0–1.0 × 10^5^	150.0	Crulhas et al. 2017 [17]
CPNT-FETs/Apt	FET	18.0–18.0 × 10^7^	18.0	Kwon et al. 2010 [32]
SiNW-FETs/Apt	FET	100.0–45.0 × 10^3^	100.0	Lee et al. 2009 [44]
SPE/PANI/CNT/Apt	DPV	0.5–10.0 × 10^6^	0.4	This work

(GE: Gold-based screen-printed electrode; GO: Graphene oxide; PLLA NP: Poly-L-lactide nanoparticle; PPy-NDFLG: Polypyrole-converted nitrogen-doped few-layer graphene; FET: Field effect transistor; Apt: Aptamer; MB-Abs: Antibody conjugated magnetic beads; nFIS: Non-Faradaic electrochemical impedance spectroscopy; SPE: Carbon-based screen-printed electrode; OMC-Aunano: Ordered mesoporous carbon-gold nanocomposite; SWV: Square wave voltammetry CPNTs: carboxylated polypyrrole nanotubes; SiNW: Silicon nanowire).

## Data Availability

Not applicable.

## Supplementary Materials

The following are available online at https://www.mdpi.com/article/10.3390/bios11040114/s1, Figure S1: Comparison of the electron charge transfer resistance on the bare and modified electrodes; Figure S2: The cyclic voltammetry spectra in redox buffer solution measured on PANI/CNT/Apt-modified electrode (Zoomed graph of Figure 5A.).
