# Supplementary material for: Highly Sensitive Electrochemical Aptasensor for Detecting the VEGF165 Tumor Marker with PANI/CNT Nanocomposites
Source: Biosensors (Basel). 2021 Apr 9;11(4):114. doi: 10.3390/bios11040114 (PMC8069203; doi:10.3390/bios11040114)
Supplement: Supplementary file 1 [file biosensors-11-00114-s001.pdf]

biosensors

Supplementary Information for Highly sensitive electrochemical aptasensor for detecting $VEGF_{165}$ tumor marker with PANI/CNT nanocomposites

Yunjeong Park [1,†], Min-Sung Hong [2,†], Woo-Hyuk Lee [2], Jung-Gu Kim [2,*], Kyunghoon Kim [1,*]

[1] School of Mechanical Engineering, Sungkyunkwan University (SKKU), Suwon 16419, Republic of Korea; djy828@skku.edu (Y.P.); kenkim@skku.edu (K.K.)
[2] School of Advanced Materials Science and Engineering, Sungkyunkwan University (SKKU), Suwon 16419, Republic of Korea; smith803@skku.edu (M.-S.H.); picohiyo@gmail.com(W.-H.L.); kimjg@skku.edu (J.-G.K.)
* Correspondence: kenkim@skku.edu (K.K.); kimjg@skku.edu (J.-G.K.)
† These authors contributed equally to this work.

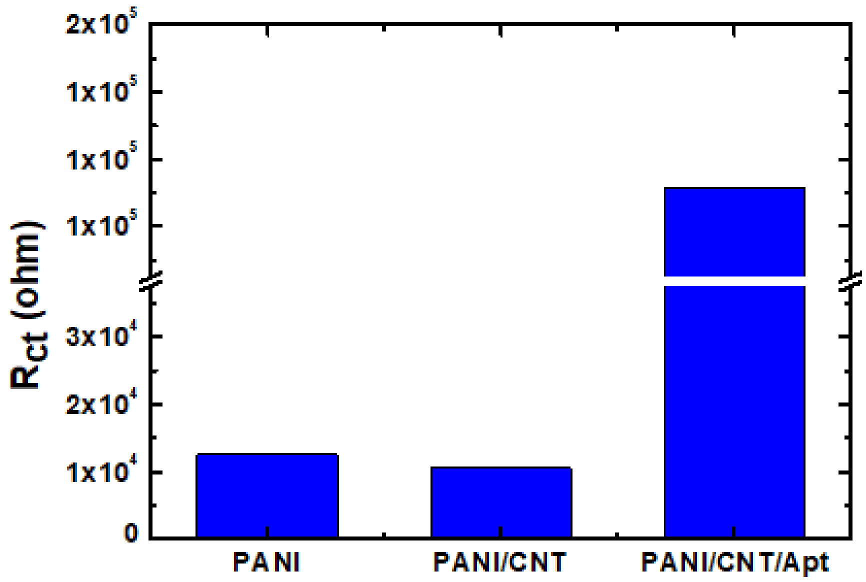

Figure S1. Comparison of the electron charge transfer resistance on the bare and modified electrodes.

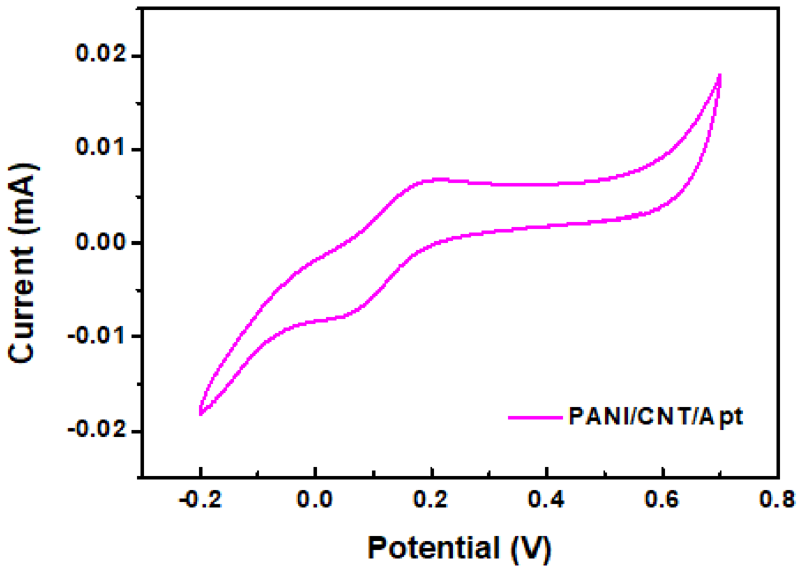

Figure S2. The cyclic votammetry spectra in redox buffer solution measured on PANI/CNT/Apt-modified electrode. (Zoomed graph of Figure 5.)
